# The biochemical and electrophysiological profiles of amniotic fluid-derived stem cells following Wnt signaling modulation cardiac differentiation

**DOI:** 10.1038/s41420-019-0143-0

**Published:** 2019-01-28

**Authors:** Yen-Wen Liu, Yi-Hsein Fang, Chi-Ting Su, Shiaw-Min Hwang, Ping-Yen Liu, Sheng-Nan Wu

**Affiliations:** 1 Division of Cardiology, Department of Internal Medicine, National Cheng Kung University Hospital, College of Medicine, National Cheng Kung University, 138 Sheng-Li Rd. North District, Tainan, 70403 Taiwan; 2 Institute of Clinical Medicine, National Cheng Kung University Hospital, College of Medicine, National Cheng Kung University, Tainan, Taiwan; 3 Division of Nephrology, Department of Internal Medicine, National Taiwan University Hospital, Yun-Lin Branch, Yun-Lin, Taiwan; 4 Bioresource Collection and Research Center, Food Industry Research and Development Institute, Hsinchu, Taiwan; 5 Institute of Basic Medical Sciences, College of Medicine, National Cheng Kung University, 1 University Rd, East District, Tainan, Taiwan; 6 Department of Physiology, College of Medicine, National Cheng Kung University, Tainan, Taiwan

## Abstract

Owing to the beneficial properties of amniotic fluid-derived stem cells (AFSCs), including pluripotency and the lack of ethical issues associated with embryonic stem cells (ESCs), they should be a promising cell source for regenerative medicine. However, how to differentiate AFSCs into contracting cardiomyocytes has not been established. In this study, a well-established, direct cardiac differentiation protocol involving the modulation of Wnt signaling was used to differentiate Oct 3/4^+^ AFSCs into cardiomyocytes. By day 14 of cardiomyocyte differentiation, these AFSCs expressed cardiac-specific genes (i.e., cardiac troponin T and myosin light chain 2v) and proteins but could not spontaneously contract. Using the patch-clamp technique, we further characterized the electrophysiological properties of human ESC-derived cardiomyocytes (hESC-CMs) and differentiated AFSCs. We used different configurations to investigate membrane potentials and ion currents in differentiated AFSCs and hESC-CMs. Under cell-attached voltage- or whole-cell current-clamp modes, we recorded spontaneous action currents (ACs) or action potentials (APs) in hESC-CMs but not in differentiated AFSCs. Compared to hESC-CMs, differentiated AFSCs showed significantly diminished activity of both BK_Ca_ and IK_Ca_ channels, which might lead to a lack of spontaneous ACs and APs in differentiated AFSCs. These results indicated that this well-established Wnt signaling modulating cardiac differentiation protocol was insufficient to induce the differentiation of functional cardiomyocytes from Oct 3/4^+^ AFSCs. Therefore, AFSC may not be an ideal candidate for cardiomyocyte differentiation.

## Introduction

After severe myocardial injury, such as myocardial infarction, the regenerative ability of mammalian hearts is very limited,^1^ which may lead to impaired cardiac systolic function, heart failure or even death. Ideally, post-infarct cardiac contractility could be restored by replacing scar tissues with functional stem cell-derived cardiomyocytes.^2^ It was reported that exogenous bone-marrow-derived c-kit^+^ hematopoietic stem cells^3^ and endogenous c-kit^+^ cardiac progenitor cells^4^ restored the infarcted myocardium, supporting the concept that stem cells may be effective for cardiac regeneration. However, several studies have shown that c-kit^+^ stem cells, including hematopoietic stem cells and cardiac progenitor cells, do not efficiently differentiate into cardiomyocytes.^5–7^ Additionally, over the last decade, hundreds of patients have received c-kit^+^ stem cell therapy, with conflicting results regarding the improvement in cardiac function.^8–13^

Human embryonic stem cells (hESCs) are pluripotent. There is no doubt that using a well-established cardiac differentiation protocol, hESCs can differentiate into contracting cardiomyocytes.^14–16^ hESC-derived cardiomyocytes (hESC-CMs) can sufficiently repair damaged cardiac tissues and result in favorable cardiac repair.^14–19^ Although cardiac regeneration using hESC-CMs is promising, significant obstacles limit their clinical application.^20^ For example, after hESC-CM transplantation, the recipients will need the life-long use of strong immunosuppressive drugs to prevent rejection of these transplanted cells^17^; nevertheless, these drugs may cause several major adverse events, such as kidney injury, serious infection, and malignancy. Additionally, the use of hESCs for research or therapy has complex social and ethical issues.

Amniotic fluid-derived stem cells (AFSCs) express the transcription factor Oct-4, indicating that they should be pluripotent.^21,22^ Importantly, owing to low major histocompatibility complex (MHC) class I antigen expression and the absence of MHC class II antigens, AFSCs may have immune privilege.^21–23^ Moreover, unlike hESCs, using AFSCs for research does not have any major ethical issues. Owing to these beneficial properties, AFSCs should be a good candidate for regenerative medicine research.^23^ Accordingly, we aimed to investigate whether AFSCs could be differentiated into contracting cardiomyocytes in vitro.

## Results

### AFSC characteristics

**Fig. 1:**
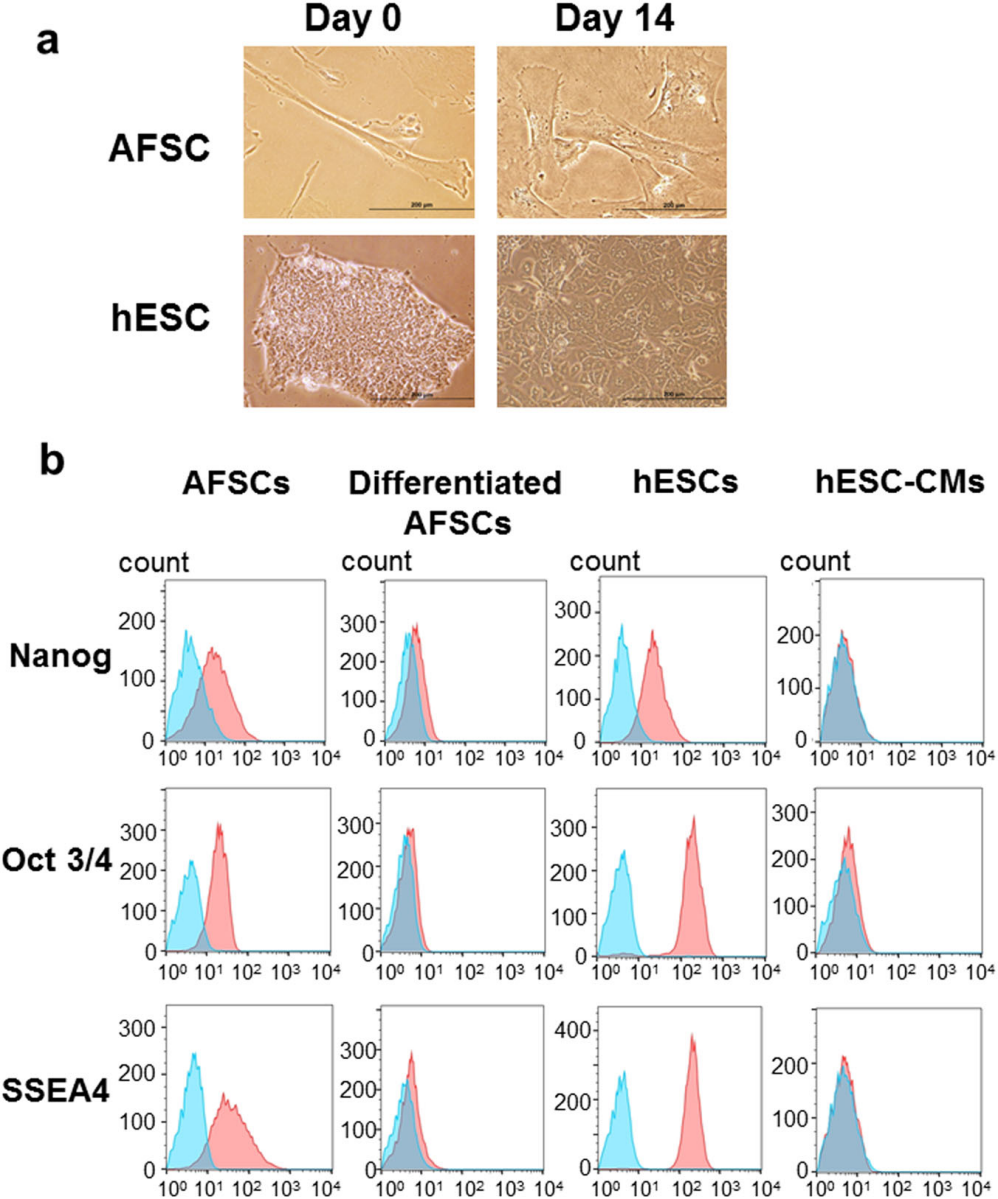
Characterization of undifferentiated and differentiated amniotic fluid-derived stem cells (AFSCs). **a** Representative images showed the appearance of undifferentiated and differentiated AFSCs, human embryonic stem cell (hESC) and hESC-derived cardiomyocytes (hESC-CMs). Undifferentiated AFSCs exhibited a heterogeneous morphology with a preponderance of fibroblastoid, mesenchymal-like cell shapes. After 14 days of differentiation, the morphology of AFSCs exhibited a rod-like appearance, different from that of human embryonic stem cell-derived cardiomyocytes. Scale bar, 200 µm. **b** Undifferentiated AFSCs and human embryonic stem cells (hESCs) expressed the pluripotent stem cell markers Nanog, Oct3/4, and SSEA4. At cardiac differentiation day 14, the expression of these 3 pluripotent stem cell markers significantly reduced in both differentiated AFSCs and hESC–derived cardiomyocytes (hESC-CMs)

Undifferentiated AFSCs predominantly exhibited a fibroblast-like morphology (Fig. 1a). Flow cytometry indicated that undifferentiated AFSCs and hESCs expressed the pluripotent stem cell markers, i.e., Nanog, Oct3/4, and SSEA4 (Table 1; Fig. 1b). At cardiac differentiation day 14, the expression of these 3 pluripotent stem cell markers significantly reduced in both differentiated AFSCs and hESC-CMs (Table 1; Fig. 1b). This finding indicated that ASFCs possessed pluripotent characteristics, similar to those of hESCs and induced pluripotent stem cells.

**Table 1:** Median fluorescence intensity (MFI) for surface markers of amniotic fluid derived stem cells and human embryonic stem cells

	MFI			
	Undifferentiated AFSCs	Differentiated AFSCs	hESCs	hESC-CMs
Nanog	648	168	632	108
Oct 3/4	551	127	5048	170
SSEA4	1512	146	5622	136
cTnT	89	1319	72	806
MLC2a	182	464	101	320
MLC2v	167	1230	111	475

*AFSC* indicated amniotic fluid derived stem cell

*cTnT* cardiac troponin T, *hESC-CMs* human embryonic stem cell derived cardiomyocytes, *MFI* median fluorescence intensity, *MLC* myosin light chain, *Oct 3/4* octamer-binding transcription factor 3/4, *SSEA4* stage-specific embryonic antigen-4

### Cardiac differentiation of AFSCs

**Fig. 2:**
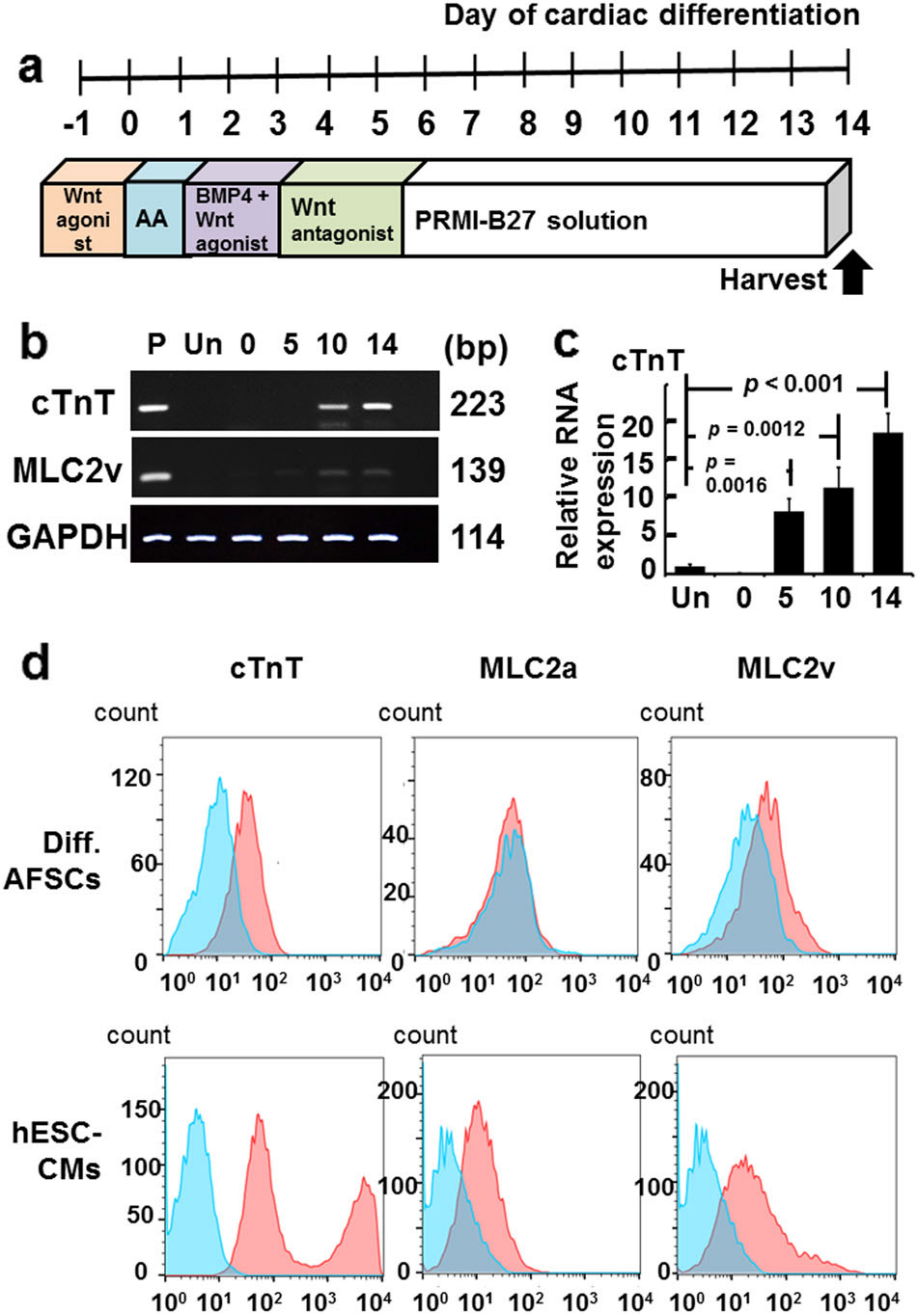
Gene and protein expression in amniotic fluid-derived stem cells (AFSCs) following cardiac differentiation. **a** Schematic representation of the cardiac differentiation protocol involving the modulation of Wnt signaling. **b** Gene expression of cardiac troponin T (cTnT) and myosin light chain 2 v (MLC2v) in those differentiating AFSCs since day 10 of cardiac differentiation was confirmed by reverse transcription-polymerase chain reaction (RT-PCR). P, positive control (human embryonic stem cell-derived cardiomyocytes, hESC-CMs); Un, undifferentiated AFSCs; 0, differentiation day 0; 5, differentiation day 5; 10, differentiation day 10; 14, differentiation day 14. **c** Relative gene expression of cTnT was measured by quantitative RT-PCR (qRT-PCR). Compared to undifferentiated AFSCs, cTnT gene expression was significantly increased since day 5 of cardiac differentiation. **d** Flow cytometry analysis of cell surface markers staining determined the percentage of cTnT-positive, MLC2a-positive, and MLC2v-positive cells after 14-day cardiac differentiation. Isotype controls are in blue and the surface markers are in red. Expression of each surface marker or isotype control was analyzed on 100,000 cells. The percentages of hESC-CMs expressing cTnT, MLC2a, and MLC2v were 93.2, 41, and 74.2%, respectively. The percentages of differentiated AFSCs expressing cTnT, MLC2a, and MLC2v were 60.2, 0.1, and 27.5%, respectively. Compared to hESC-CMs, the differentiated AFSCs did not show a typical bimodal distribution of cardiac differentiation

Using the direct cardiac differentiation protocol based on the Wnt signaling pathway (Fig. 2a), differentiated AFSCs were elongated and larger in size than undifferentiated cells (Fig. 1a). During the differentiation period, significant changes in cardiac gene expression, i.e., positive expression of both cardiac troponin T (cTnT) and myosin light chain (MLC) 2v, were observed since differentiation day 10 (Fig. 2b). A quantitative reverse transcription polymerase chain reaction (qRT-PCR) analysis of cTnT expression was performed on days 0, 5, 10, and 14. Relative cTnT gene expression was significantly higher on days 5, 10, and 14 in differentiated ASFCs than in undifferentiated cells (undifferentiated AFS cells: 1 ± 0.25; day 0: 0.13 ± 0.01; day 5: 8.24 ± 1.67; day 10: 11.38 ± 2.7; day 14: 18.67 ± 2.52; undifferentiation vs. day 5: *p* = 0.0016; undifferentiation vs. day 10: *p* = 0.0012; undifferentiation vs. day 14: *p* < 0.001; Fig. 2c).

At differentiation day 14, we performed flow cytometry using both differentiated AFSCs and hESC-CMs to evaluate expression changes in cardiac-specific sarcomeric proteins (i.e., cTnT, MLC2a, and MLC2v) (Fig. 2d). These differentiated cells were not positive for MLC2a, indicating that they were not atrial cardiomyocytes. Compared to undifferentiated AFSCs, these differentiated AFSCs had significantly higher expression levels of ventricular cardiomyocyte markers, i.e., cTnT and MLC2v (Table 1; Fig. 2d). The percentages of differentiated AFSCs expressing cTnT and MLC2v were 60.2 and 27.5%, respectively. Although the percentage of differentiated AFSCs expressing cTnT was 60.2%, a typical bimodal distribution of cardiomyocytes was not detected (Fig. 2d), indicating that these differentiated AFSCs were not true ventricular cardiomyocytes or were immature ventricular cardiomyocytes. Because the expression pattern of MLC2v may provide information regarding the maturity of differentiated cells,^24,25^ we used flow cytometry to evaluate MLC2v expression and found a low percentage of MLC2v expression in differentiated AFSCs.

**Fig. 3:**
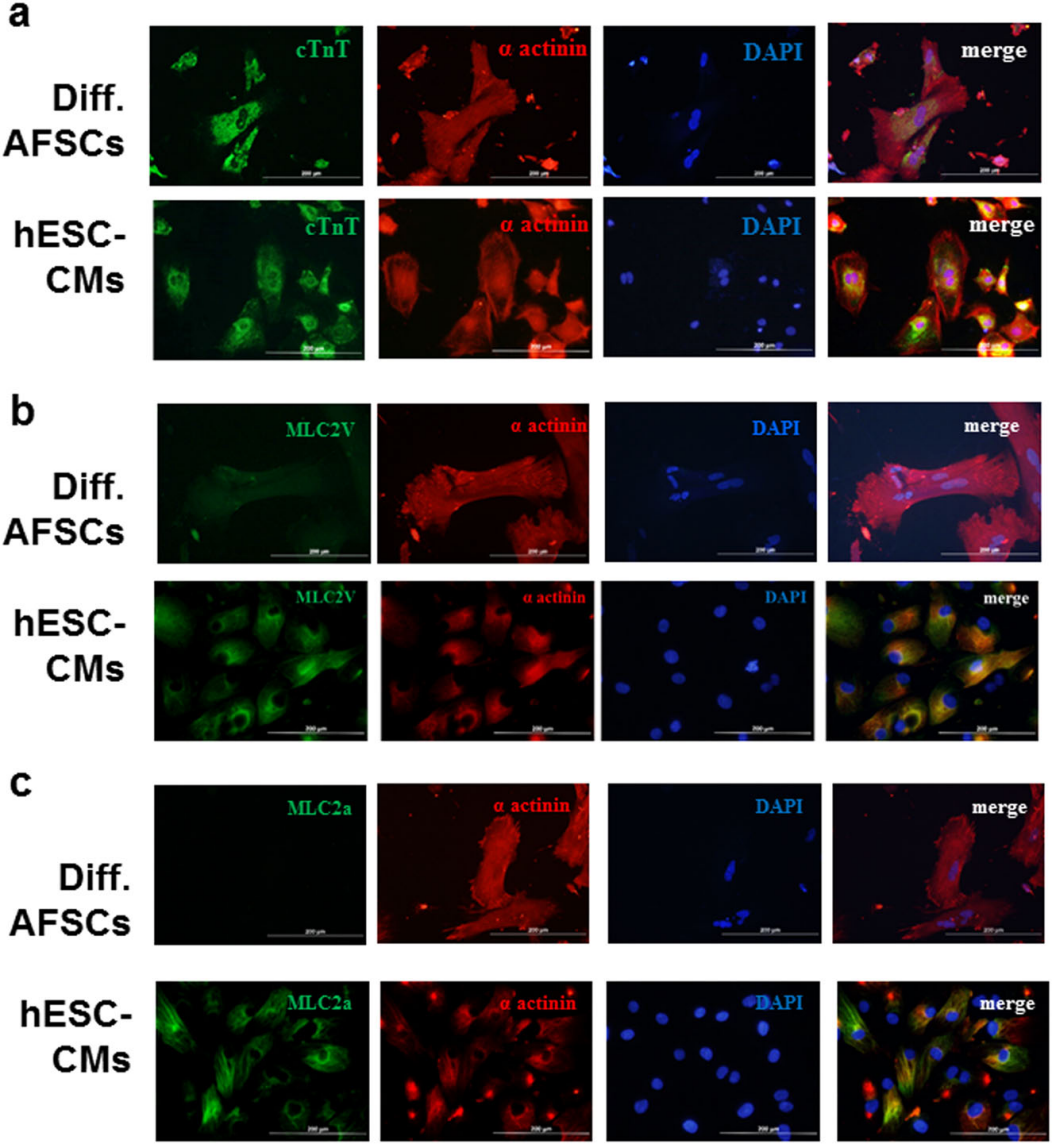
Immunofluorescence staining in amniotic fluid derived stem cells (AFSCs) and human embryonic stem cells (hESCs) following cardiac differentiation. Immunostaining for **a** cardiac troponin T (cTnT), **b** myosin light chain (MLC)2v, and **c** MLC2a detection in differentiated AFSCs and hESC-derived cardiomyocytes (hESC-CMs). The differentiated AFSCs expressed cTnT and MLC2v but not MLC2a. This finding was compatible with the gene expression results. These results suggested that AFSCs after 14 days of cardiac differentiation were cardiac ventricular-like cells. Scale bars represent 200 µm

Moreover, we performed and quantified immunofluorescence staining on day 14 of cardiac differentiation (Table 2; Fig. 3). Obvious cTnT expression was observed in differentiated AFSCs and hESC-CMs (Fig. 3a), but low expression of MLC2v was noted in differentiated AFSCs (Fig. 3b). Furthermore, differentiated AFSCs did not express MLC2a (Fig. 3c). These immunofluorescence staining results were compatible with the flow cytometry results. Our results indicated that the differentiated AFSCs were not atrial cardiomyocytes and were likely immature ventricular cardiomyocytes or cardiomyocyte-like cells.

**Table 2:** Quantification of the fluorescence intensity for immunofluorescent staining in differentiated amniotic fluid derived stem cells and human embryonic stem cell-derived cardiomyocytes

	Fluorescence intensity	
	Differentiated AFSCs	hESC-CMs
cTnT	95.94 ± 5.82	83.27 ± 3.43
MLC2a	N/A	81.84 ± 5.13
MLC2v	11.87 ± 2.56	72.11 ± 2.25

*AFSC* indicated amniotic fluid derived stem cell. Data were expressed as mean ± SEM

*cTnT* cardiac troponin T, *hESC-CMs* human embryonic stem cell-derived cardiomyocytes, *MLC* myosin light chain, *N/A* not available

### Characterization of the electrophysiological properties of differentiated AFSCs and hESC-CMs

Although differentiated AFSCs expressed cardiac-specific markers (i.e., cTnT and MLC2v), they did not contract spontaneously. Therefore, it is necessary to identify the difference in electrophysiological characteristics between differentiated AFSCs and hESC-CMs. By using whole-cell current-clamp recordings, we evaluated membrane potential in differentiated AFSCs and hESC-CMs. A periodic change in membrane potential was clearly detected in hESC-CMs (Supplementary figure 1) but not in differentiated AFSCs. The hESC-CM amplitude, firing frequency, and initial rate of increase in spontaneous APs were 64 ± 4 mV, 0.29 ± 0.03 Hz, and 0.72 mV/ms (*n* = 9), respectively. To study spontaneous ACs in hESC-CMs, we utilized cell-attached current recordings.^26,27^ The resting membrane potential of these hESC-CMs was approximately −65 mV. Biphasic current waveforms (i.e., ACs) induced across the membrane patch by intracellular APs were observed in hESC-CMs (Supplementary figure 2) but not in differentiated AFSCs. These currents with irregular amplitudes are thought to be electrical manifestations of APs as cell-attached voltage-clamp recordings are achieved. The spontaneous ACs of hESC-CMs with a firing frequency of 0.36 ± 0.01 Hz (*n* = 8) were measured. Notably, the emergence of ACs appears when a spike in the downward deflection occurs.

### Characterization of large-conductance Ca^2+^-activated K^+^ (BK_Ca_) channel activity in AFSCs

**Fig. 4:**
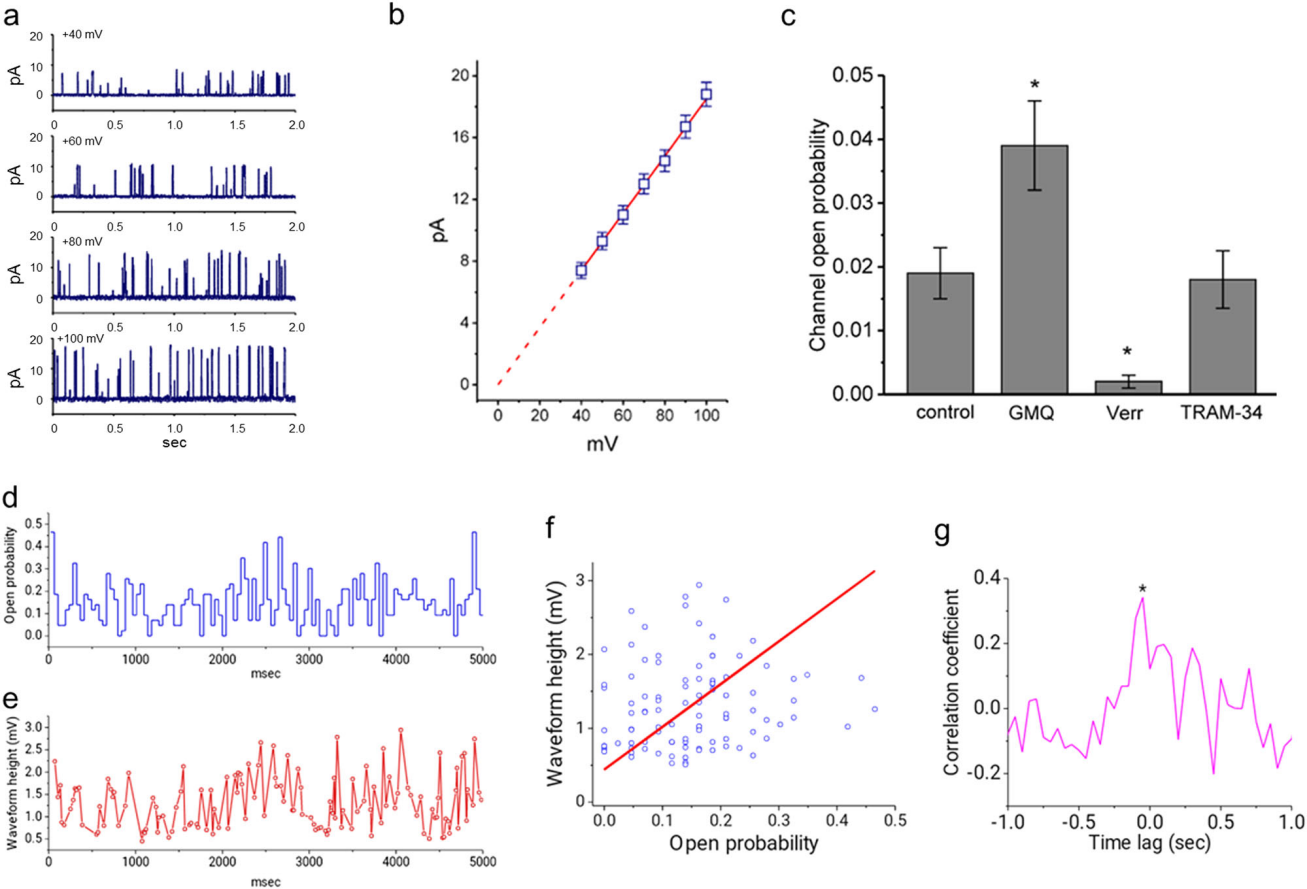
Characterization of BK_Ca_-channel activity recorded from undifferentiated amniotic fluid-derived stem cells (AFSCs). Cells were bathed in a high K^+^ solution containing 0.1 μM Ca^2+^. **a** Original BK_Ca_ currents for different levels of membrane potential (indicated on the upper part of each trace). The upward deflection indicates an opening event of the BK_Ca_ channel. **b** Based on the *I*–*V* relationship of BK_Ca_ channels, the single-channel conductance in these undifferentiated AFSCs was 185 ± 4 pS (*n* = 11). Note that the dashed line indicates the reverse potential (i.e., 0 mV). **c** This bar graph summarized the effects of GMQ (10 μM), verruculogen (Verr, 1 μM), or TRAM-34 (1 μM) on the BK_Ca_-channel open probability measured at + 60 mV (mean ± S.E.M.; *n* = 9 for each bar). The addition of 10 μM GMQ significantly increased the probability of channel openings (control vs. GMQ: 0.019 ± 0.004 vs. 0.039 ± 0.007, *n* = 9/group, *p* = 0.008), whereas 1 μM verruculogen effectively decreased open BK_Ca_ channels (control *vs*. verruculogen: 0.019 ± 0.004 vs. 0.002 ± 0.001, *n* = 9/group, *p* = 0.004). **d**–**g** Each large current downward deflection, with an amplitude of 3.1 ± 0.1 pA (*n* = 12), indicated the opening of a single BK_Ca_ channel. We simultaneously measured the open channel probability (**d**) and the waveform height (**e**) of the membrane potential in differentiated AFSCs. Each circled symbol in **e** indicates detection of the depolarizing waveform. **f** The probability of channel openings tended to be positively correlated with changes in the amplitude of depolarizing waveforms. The slope between the open channel probability and the waveform height was 0.44. **g** This graph showed the cross-correlation plot (i.e., correlation coefficient versus time lag) with a time lag of approximate 5.0 ms (indicated by an asterisk). ^*^Significantly different from the control (*p* *<* 0.05)

Because we were unable to detect the APs and ACs of differentiated AFSCs, we were interested in ion channel expression. Thus, we characterized membrane ion currents in undifferentiated and differentiated AFSCs. These AFSCs were immersed in a high K^+^ solution (145 mM) containing 0.1 μM Ca^2+^. When inside-out current recordings were established, BK_Ca_ channel activity was observed at different levels of holding potential (Fig. 4a). Unexpectedly, in undifferentiated AFSCs, but not in differentiated AFSCs, we detected high activity of BK_Ca_ channels, as previously reported in human cardiac fibroblasts.^28^ Based on the *I*–*V* relationship of these channels (Fig. 4b), the single-channel conductance in these undifferentiated AFSCs was 185 ± 4 pS (*n* = 11). Moreover, the addition of 10 μM 2-guanidine-4-methylquinazoline (GMQ) significantly increased the probability of channel openings (control vs. GMQ: 0.019 ± 0.004 vs. 0.039 ± 0.007, *n* = 9/group, *p* = 0.008), whereas 1 μM verruculogen effectively decreased open BK_Ca_ channels (control *vs*. verruculogen: 0.019 ± 0.004 vs. 0.002 ± 0.001, *n* = 9/group, *p* = 0.004) (Fig. 4c). However, neither GMQ nor verruculogen was capable of modifying the single-channel conductance of these channels.

Each large current downward deflection, with an amplitude of 3.1 ± 0.1 pA (*n* = 12), indicated the opening of a single BK_Ca_ channel. Notably, the current deflection coincided with the emergence of a depolarizing waveform, observed as an upward deflection. A change in the depolarizing waveform may result from the opening trajectory of the channel, which was distorted with a current relaxation of approximately 14 ms. When the open state probability of a single BK_Ca_ channel was derived and plotted over time (Fig. 4d, e), the probability of channel openings tended to be positively correlated with changes in the amplitude of depolarizing waveforms (Fig. 4f). The time lag was 4.9 ± 0.2 ms (*n* = 12) (Fig. 4g).

### Characterization of intermediate-conductance Ca^2+^-activated K^+^ (IK_Ca_) channel activity in AFSCs

**Fig. 5:**
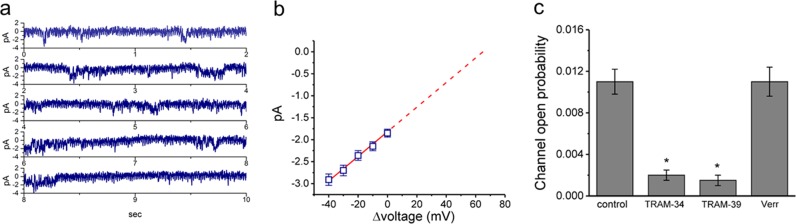
Characterization of IK_Ca_-channel activity in undifferentiated amniotic fluid-derived stem cells (AFSCs). **a** Original IK_Ca_-channel currents were obtained at 0 mV relative to the bath. The downward deflection indicates the opening event of the undifferentiated AFSC IK_Ca_ channel. **b** A single IK_Ca_-channel amplitude (*n* = 9 for each point) was plotted as a function of potential (i.e., Δvoltage). Notably, as the patch potential is the sum of the resting potential and the pipette potential, these inward currents reverse at approximately +65 mV. As different levels of membrane potential were applied to the undifferentiated AFSCs, the *I*–*V* relationship for IK_Ca_ channels (i.e., single-channel amplitude versus Δvoltage) was established. The single-channel conductance was 28.1 ± 1.1 pS (*n* = 9). Moreover, the dashed line indicates the reversal of potential (i.e., + 65 mV). **c** The effects of TRAM-34 (1 μM), TRAM-39 (1 μM), or verruculogen (Verr, 1 μM) on the probability of IK_Ca_-channel openings were investigated (*n* = 9 for each bar). The opening events of IK_Ca_ channels were measured at 0 mV relative to the bath solution. The addition of either TRAM-34 (1 μM) or TRAM-39 (1 μM) significantly decreased the probability of IK_Ca_-channel openings (control vs. TRAM-39: 0.011 ± 0.001 vs. 0.0015 ± 0.0005, *p* *=* 0.001), while verruculogen (1 μM) did not have any effect (control vs. verruculogen: 0.011 ± 0.001 vs. 0.0011 ± 0.0014, *p* = 0.06) ^*^Significantly different from the control (*p* *<* 0.05). 1-[(2-Chlorophenyl)diphenylmethyl]-^1^H-pyrazole indicates TRAM-34; 2-chloro-α,α-diphenyl benzeneacetonitrile, TRAM-39

When the membrane was maintained at 0 mV relative to the bath solution, we could detect the IK_Ca_ channel activity of undifferentiated AFSCs (Fig. 5a). Again, the IK_Ca_ activity of differentiated AFSCs was not detected. As different levels of membrane potential were applied to the undifferentiated AFSCs, the *I*–*V* relationship for IK_Ca_ channels (i.e., single-channel amplitude versus Δvoltage) was established (Fig. 5b). The single-channel conductance was 28.1 ± 1.1 pS (*n* = 9). Moreover, the addition of either 1-[(2-chlorophenyl)diphenylmethyl]-^1^H-pyrazole (TRAM-34) (1 μM) or 2-chloro-α,α-diphenyl benzeneacetonitrile (TRAM-39) (1 μM) significantly decreased the probability of IK_Ca_-channel openings (control *vs*. TRAM-39: 0.011 ± 0.001 vs. 0.0015 ± 0.0005, *n* = 9/group, *p* = 0.001), while verruculogen (1 μM) did not have any effect (control vs. verruculogen: 0.011 ± 0.001 vs. 0.0011 ± 0.0014, *n* = 9/group, *p* = 0.06) (Fig. 5c). When the pipette solution included 1 μM chlorotoxin (a Cl^-^ channel blocker, a gift from Dr. Woei-Jer Chuang, Department of Biochemistry, College of Medicine, National Cheng Kung University, Tainan, Taiwan), the IK_Ca_ channel remained functionally active and was sensitive to suppression by TRAM-34, but not by verruculogen.

### Characterization of voltage-gated L-type Ca^2+^ current (*I*_Ca,L_) in differentiated AFSCs

**Fig. 6:**
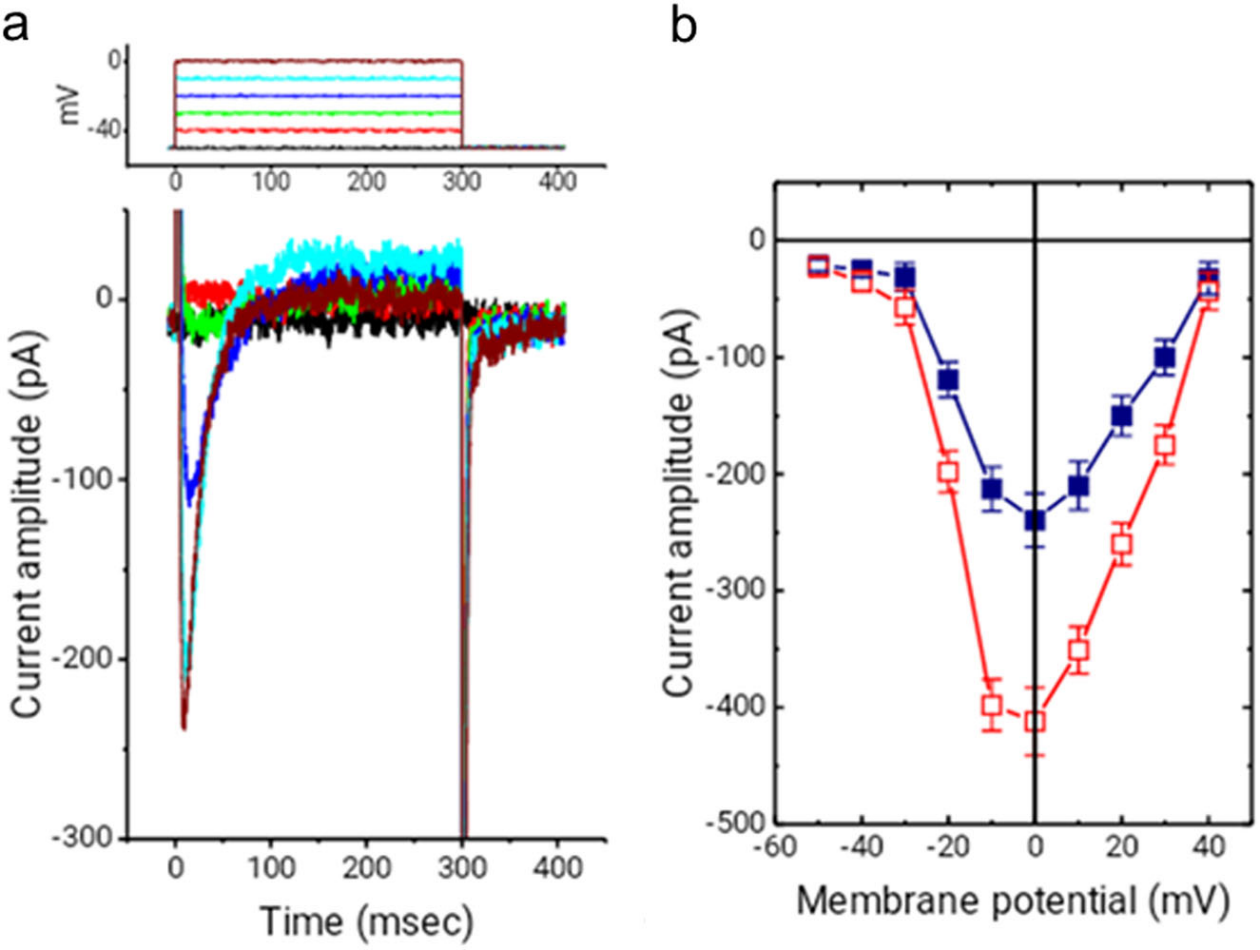
Properties of the voltage-gated L-type Ca^2+^ current (*I*_Ca,L_) recorded from differentiated amniotic fluid-derived stem cells (AFSCs). The differentiated AFSCs were immersed in a bath solution containing 1 μM tetrodotoxin and 10 μM tetraethylammonium chloride, and the recording pipettes were filled with a Cs^+^-containing solution. **a** Superimposed current traces elicited in response to depolarizing pulses (the upper part). We detected the presence of *I*_Ca,L_ in differentiated AFSCs and the properties of *I*_Na_ caused by rapid membrane depolarization was clearly identified. **b** Average *I–V* relations of peak *I*_Ca,L_ in the absence (■) or presence (□) of 0.1 μM isoproterenol (*n* = 8 for each point). Cells exposed to isoproterenol had an increasing peak *I*_Ca,L_ but there was no significant change in the overall *I–V* relationship of *I*_Ca,L_ or the current inactivation process when differentiated AFSCs were treated with isoproterenol

Although the activities of BK_Ca_ and IK_Ca_ channels were significantly reduced in differentiated AFSCs, we detected the presence of *I*_Ca,L_ in differentiated AFSCs (Fig. 6a). Additionally, the properties of *I*_Na_ caused by rapid membrane depolarization could be clearly identified. The depolarizing step also gives rise to *I*_Ca,L_.^29^ Although 0.1 μM isoproterenol (Sigma-Aldrich, St. Louis, MA, USA) could effectively increase the peak *I*_Ca,L_ amplitude (Fig. 6b), there was no significant change in the overall *I–V* relationship of *I*_Ca,L_ or the current inactivation process when differentiated AFSCs were treated with isoproterenol. These differentiated AFSCs expressed cardiac-specific biomarkers and had *I*_Na_ and *I*_Ca,L_ activity but these cells did not have significant BK_Ca_ and IK_Ca_ channel activity, which may explain why these differentiated AFSCs had cardiac sarcomeric protein expression but could not spontaneously contract.

## Discussion

In this study, we showed that Oct 3/4^+^ AFSC differentiation into functional cardiomyocytes was not possible using a well-established direct cardiac differentiation protocol, despite the expression of cardiac-specific genes and proteins in differentiated AFSCs. Connexin 43 has been detected in the cell junctions between cardiac-differentiated AFSCs.^30,31^ However, we found that these differentiated AFSCs could not spontaneously contract. We further characterized the electrophysiological properties of hESC-CMs and differentiated AFSCs using various configurations of the patch-clamp technique. Under cell-attached voltage- or whole-cell current-clamp modes, we recorded spontaneous ACs or APs in hESC-CMs but not in differentiated AFSCs. Moreover, both BK_Ca_ and IK_Ca_ channel activity were detected in hESC-CMs but were significantly decreased in differentiated AFSCs.

In previous studies, c-kit^+^ Oct 3/4^-^ AFSCs did not efficiently differentiate into cardiomyocytes.^30,32^ Oct 3/4 is the gatekeeper for stem cell pluripotency^33–36^ and is expected to be an important factor for cardiac differentiation.^37–39^ In this study, AFSCs expressed the transcription factors Oct 3/4, Nanog, and SSEA, indicating self-renewal ability and stem cell pluripotency. Therefore, using a well-defined direct cardiomyocyte differentiation protocol, we proposed that Oct 3/4^+^AFSCs could differentiate into cardiomyocytes. Indeed, the expression of cardiac-specific genes (i.e., cTnT and MLC2v) could be detected on differentiation day 5 and increased significantly thereafter (Fig. 2a, b). It is worth mentioning that compared to hESC-CMs, although day-14 differentiated AFSCs expressed cTnT and MLC2v, a bimodal distribution of cTnT expression was not observed (Fig. 2c) and only a relatively small percentage of differentiated AFSCs were positive for MLC2v. Importantly, no spontaneous contraction was observed during the 2-week differentiation period. Therefore, our results indicate that these differentiated AFSCs might be ventricular-like cells but are not real ventricular cardiomyocytes.

Because these differentiated AFSCs expressed connexin 43 but could not spontaneously contract, we were interested in their electrophysiological characteristics. Based on patch-clamp electrophysiological studies, hESC-CMs showed spontaneous ACs or APs, which could be suppressed by 10 μM ranolazine (Supplemental Figure 2). When hESC-CMs were treated with tefluthrin (10 μM), a synthetic type-I pyrethroid, there were increases in the amplitude and frequency of ACs.

Moreover, these hESC-CMs displayed periodic rhythms (Supplemental Figure 1) over several minutes in vitro; accordingly, it is important to use an electrophysiological system that does not interfere with cell behavior to record the firing patterns of these contracting cells. Cell-attached current-clamp or voltage-clamp recordings may accomplish this objective with minimal effort and without causing significant damage to cells.^26,27,40^ The openings of single hESC-CM BK_Ca_ channels could potentially trigger fluctuations in membrane potential.^41^ Based on these results, we proposed that the random opening and closure of BK_Ca_ channels could lead to the stochastic triggering of depolarizing waveforms of cardiomyocytes. In hESC-CMs with high input resistance, current relaxation resulting from the opening of BK_Ca_ channels can depolarize cells. After the temporal or spatial summation of depolarizing currents, APs were generated. hESC-CMs exhibited BK_Ca_ activity and were electrically coupled. However, the BK_Ca_ and IK_Ca_ channel activity of differentiated AFSCs was significantly diminished, though these differentiated AFSCs exhibited *I*_Na_ and *I*_Ca,L_ activity. The functional expression of BK_Ca_ and IK_Ca_ channels was reduced during differentiation. This novel finding may explain, in part, why differentiated AFSCs expressed cardiac-specific markers but could not spontaneously contract.

## Conclusion

AFSCs are viewed as a promising cell source for regenerative medicine. However, our results showed that Wnt signaling modulation could not efficiently induce functional cardiomyocyte differentiation from AFSCs. Differentiated AFSCs expressed cardiac-specific genes and proteins but did not spontaneously contract. Our electrophysiological analysis revealed that the decreased activity of both BK_Ca_ and IK_Ca_ channels in differentiated AFSCs might lead to a lack of spontaneous ACs and APs, explaining the absence of spontaneous contraction.

## Methods and materials

### Cell culture conditions

Under a 5% CO_2_ atmosphere at 37 °C, both human AFSCs (a gift from Dr. Shiaw-Min Hwang, National Health Research Institute Cell Bank, Bioresource Collection and Research Center, Food Industry Research and Development Institute, Hsinchu, Taiwan) and hESCs (RUES2 cells, a gift from Dr. Patrick C.H. Hsieh and Dr. Jean Lu, Institute of Biomedical Sciences, Academia Sinica, Taipei, Taiwan) were maintained in α-Minimum Essential Media (MEM) (11900-024; Gibco, Waltham, MA, USA) containing 15% fetal bovine serum (FBS, SH30087.03; HyClone, Boston, MA, USA), 1% glutamine (GlutaMAX Supplement, 35050061; Gibco), and 1% penicillin/streptomycin (15140148; Gibco) for further in vitro experiments.^23^ The experimental protocol was approved by the Institutional Review Boards of National Cheng Kung University Hospital, Tainan, Taiwan (IRB No. A-EX-105-034).

Undifferentiated human AFSCs were expanded using α-MEM supplemented with 4 ng/ml basic fibroblast growth factor (bFGF, 233-FB; R&D Systems, Minneapolis, MN, USA). hESCs were used as a positive control for cardiomyocyte differentiation experiments. Using a well-established direct differentiation protocol (Murry Lab, Institute for Stem Cell and Regenerative Medicine, University of Washington, Seattle, WA, USA)^5,14–16^, the differentiation of both AFSCs and hESCs into cardiomyocytes was evaluated (Fig. 1a; Table 1).

### Immunocytochemical staining

Differentiated AFSCs and hESCs were fixed in 4% paraformaldehyde and then washed with phosphate-buffered saline (PBS). Fixed cells were treated with 1.5% normal goat serum (X0907; Dako, Santa Clara, CA, USA) for 1 h at room temperature (22–26 °C) and incubated with primary antibodies overnight at 4°C. Antibodies (Supplemental Table 1) used in this study included mouse anti-α-actinin, mouse anti-myosin light chain (MLC)2v, and mouse anti-cardiac troponin T (cTnT). After rinsing with PBS, samples were incubated with secondary antibodies (Goat anti mouse Alexa Fluor 488, ab150117 and Goat anti rabbit Alexa Fluor 568, ab175471; Abcam, Cambridge, UK). Fluorescent images were acquired (BX51; OLYMPUS, Tokyo, Japan). The confocal images were analyzed and quantified by using the Adobe Photoshop.

### Flow cytometry

At day 14 of cardiac differentiation, the dissociated cells were stained for cTnT, MLC2a, and MLC2v. Fluorescence characterization and analyses were performed using a BD FACS Canto II (BD Biosciences, Franklin Lakes, NJ, USA).

### Quantitative reverse transcription polymerase chain reaction (qRT-PCR)

RNA was extracted from undifferentiated/differentiated AFSCs and hESC-CMs and reverse-transcribed into cDNA. qRT-PCR was performed using all samples with oligonucleotide primers (Supplemental Table 2) in triplicate. Target gene expression levels were normalized against *GAPDH* expression. Gene expression was quantified with SYBR Green Master Mix (4309155; Applied Biosystems, Waltham, MA, USA) and detected using Applied Biosystems Step One Plus. Log2-fold changes were evaluated by the ΔΔCT method, using values for the undifferentiated AFSC group as a benchmark.

### Chemicals and solutions for electrophysiological analyses

Normal Tyrode’s solution was used as the bath solution for the electrophysiological analysis. To measure macroscopic K^+^ currents as well as changes in membrane potential and to eliminate contamination by Cl^-^ currents, a patch pipette was filled with a solution containing 130 mM K-aspartate, 20 mM KCl, 1 mM KH_2_PO_4_, 1 mM MgCl_2_, 3 mM Na_2_ATP, 0.1 mM Na_2_GTP, 0.1 mM EGTA, and 5 mM HEPES-KOH buffer, pH 7.2. To record the voltage-gated Na^+^ current (*I*_Na_) and L-type Ca^2+^ current (*I*_Ca,L_), equimolar Cs^+^ ions were replaced with K^+^ ions in the pipette solution and CsOH was used to adjust the pH to 7.2. To measure BK_Ca_-channel activity, a high K^+^ bathing solution was used (145 mM KCl, 0.53 mM MgCl_2_, and 5 mM HEPES-KOH buffer, pH 7.4) and the recording pipette was filled with a solution containing 145 mM KCl, 2 mM MgCl_2_ and 5 mM HEPES-KOH buffer, pH 7.2.

### Electrophysiological measurements

Shortly prior to each experiment, a 1% trypsin/EDTA solution was used to dissociate cells and an aliquot of the cell suspension was placed on a recording chamber that was tightly affixed to an inverted fluorescence microscope stage (CKX-41; Olympus). The microscope was equipped with a digital video system (DCR-TRV30; Sony, Tokyo, Japan) with a maximum magnification of 1500×. During the recordings, cells were immersed at room temperature in normal Tyrode’s solution containing 1.8 mM CaCl_2_ and were identified visually using a microscope equipped with differential interference contrast optics and a 40× objective lens. The electrodes were fabricated from Kimax-51 capillaries with an external diameter of 1.5 mm (34500; Kimble Chase, Vineland, NJ, USA). These electrodes had a tip resistance of 3–5 MΩ. Additionally, an anti-vibration air table was used to avoid mechanical noise. Using an RK-400 (Bio-Logic, Claix, France) or Axopatch 200B (Molecular Devices, Sunnyvale, CA, USA) amplifier, patch-clamp recording experiments were performed in the cell-attached, inside-out, or whole-cell configuration.^42^ The recordings were commonly achieved by advancing a pipette until it was observed to distort the cell; next, negative pressure by gentle suction of the cell membrane was applied to form a high resistance seal.

Using cell-attached clamp recording, action currents (ACs) and action potentials (APs) of hESC-CMs and undifferentiated and differentiated AFSCs were measured.^26,27,40^ The amplitude and frequency of ACs were evaluated using Mini-Analysis (Synaptosoft, Leonia, NJ, USA). During voltage-clamp recordings, the potential was maintained at approximately −65 mV. AC measurements enabled the quantification of AP frequency. AC waveforms were mainly due to the capacitive current when a cell fired an AP and emerged as a brief spike in the downward deflection.

Signals were obtained using a digital oscilloscope (model 1602; Gould, Chandler, AZ, USA). Data were stored in a laptop at 10 kHz using an acquisition interface (Digidata-1440; Molecular Devices) and analyzed using either pCLAMP 10.2 (Molecular Devices) or 64-bit OriginPro 2016 (OriginLab, Northampton, MA, USA). Through digital-to-analog conversion, the voltage-step protocol generated by pCLAMP was implemented to determine the *I–V* relationships for membrane ion currents, such as *I*_Na_ or *I*_Ca,L_. The activation or inactivation time constants of *I*_Na_ or *I*_Ca,L_ elicited by membrane depolarizations were appropriately estimated by fitting current trajectories to a single or double exponential function with the non-linear least-squares procedure.

### Single-channel analyses

Ion channel activity was analyzed using pCLAMP 10.2. Multi-Gaussian adjustments of the amplitude distributions among channels were used to determine the channel opening events. The number of active channels was defined as the maximum number of simultaneously open channels when the maximal channel open probability was achieved. The open state probabilities were computed using an iterative process to minimize estimated *χ*^2^ values. Single-channel conductance, such as the large-conductance Ca^2+^-activated K^+^ channel (BK_Ca_) or intermediate-conductance Ca^2+^-activated K^+^ channel (IK_Ca_), was determined by linear regression using mean values of current amplitudes at different potential levels. Furthermore, we added 10 μM 2-guanidine-4-methylquinazoline (GMQ) (Tocris, Bristol, UK), an opener of BK_Ca_ channels, and 1 μM verruculogen (Alomone Labs, Jerusalem, Israel), an inhibitor of BK_Ca_ channels, to manipulate the activity of BK_Ca_ channels. To complete the electrophysiological studies of IK_Ca_, we added 1-[(2-chlorophenyl)diphenylmethyl]-^1^H-pyrazole (TRAM-34) (1 μM; Tocris), 2-chloro-α,α-diphenyl benzeneacetonitrile (TRAM-39) (1 μM; Tocris) and verruculogen (1 μM).

### Statistical analyses

Continuous data are expressed as means ± standard error of mean (S.E.M.). Comparisons were conducted using Student’s *t*-tests and nonparametric Kruskal–Wallis tests when data were and were not normally distributed, respectively. A two-sided *p-*value of <0.05 was regarded as statistically significant. Statistical analyses were performed using SPSS version 22.0 (IBM Corp., Armonk, NY, USA).

## Supplementary information

Supplemental figures
