# Supplementary material for: The biochemical and electrophysiological profiles of amniotic fluid-derived stem cells following Wnt signaling modulation cardiac differentiation
Source: Cell Death Discov. 2019 Jan 28;5:59. doi: 10.1038/s41420-019-0143-0 (PMC6349909; doi:10.1038/s41420-019-0143-0)

Supplemental table 1. Information of culture medium and supplemental reagents for cardiac differentiation.

| Medium / chemical | Company | Catalog number |
| --- | --- | --- |
| α-MEM medium | Gibco, Invitrogen | 11900024 |
| Fetal bovine serum | HyClone | SH30070.03 |
| RPMI | Gibco, Invitrogen | 11875119 |
| B27 (minus insulin) | Gibco, Invitrogen | A18956-01 |
| B27 (plus insulin) | Gibco, Invitrogen | 17504-044 |
| FGF basic | Peprotech | AF-100-18B |
| CHIR 99021 | Tocris | 4423 |
| BMP4 | R&D | 314-BP |
| Activin A | R&D | 338-AC |
| Xav 939 | Sigma | X3004 |

Supplemental table 2. The primers of PCR and produce size

| Gene (marker) | Primer sequence | Size (bp) |
| --- | --- | --- |
| cTnT | F: TGGAGAGAGAGTGGACTTTGA  R: TCTC TTCAGCCAGG CGGTT | 223 |
| MLC2V | F: ACGAGTGAACGTGAAAAATG  R: CTTGAATGCGTTGAGAATGG | 139 |
| GAPDH | F:  TCCAAAATCAAGTGGGGCGA  R: TGATGACCCTTTTGGCTCCC | 114 |

Supplemental figure 1. Characterization of spontaneous action potential (APs) in human embryonic stem cell-derived cardiomyocytes (hESC-CMs). Cells were bathed normal Tyrode’s solution containing 1.8 mM CaCl_2_, the recording pipettes were filled with K^+^-containing solution, and whole-cell current-clamp recordings were made in these experiment. Original potential trace obtained from hESC-CMs.


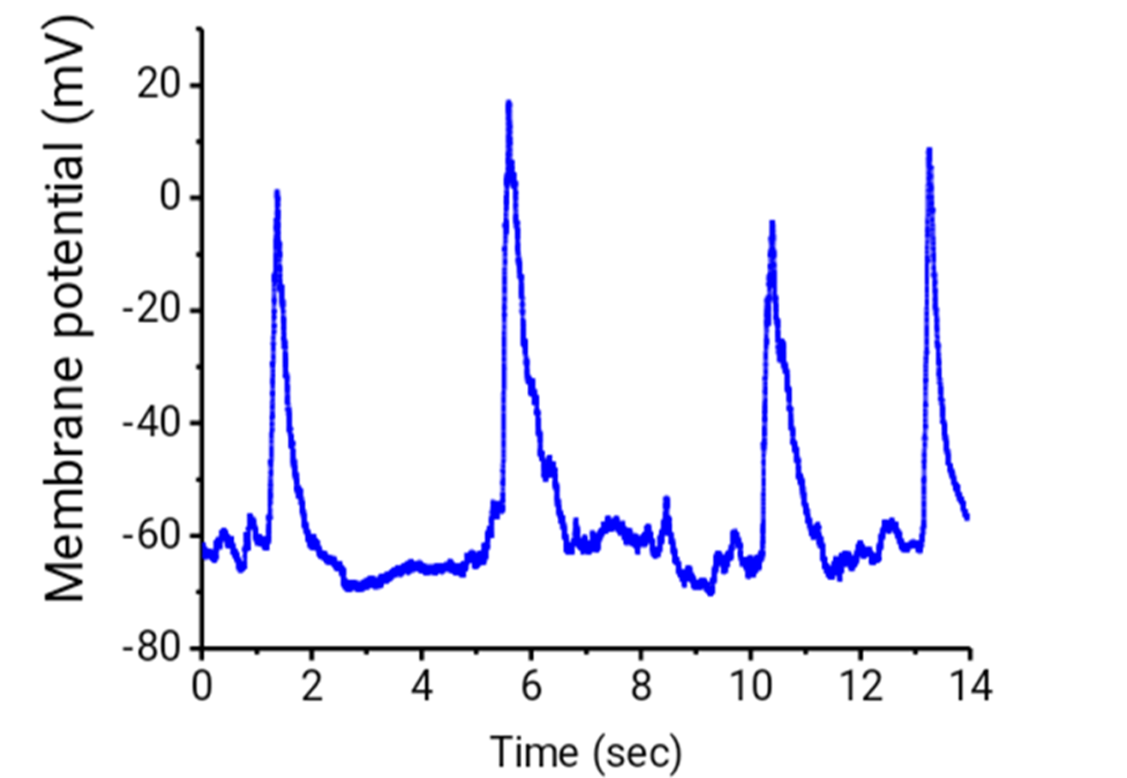


Supplemental figure 2. Characterization of spontaneous action currents (ACs) in RUES-2 cells. Cell-attached voltage-clamp recordings were conducted in cells bathed in normal Tyrode’s solution. The potential across the patch was set at the level of the resting membrane potential of the cells (around -65 mV). In **(A)**, current traces were taken in the absence (a) and presence (b) of 10 μM ranolazine. AC waveforms were notably induced cross the patch by intracellular APs. **(B)** Summary of the data showing effect of ranolazine (10 μM) and ranolazine (10 μM) plus tefluthrin (10 μM) on the frequency of ACs in RUES-2 cells (mean±SEM; n=8 for each bar). ^*^Significantly different from control (*P*<0.05) and ^**^significantly different from ranolazine (10 μM) alone group (*P*<0.05).


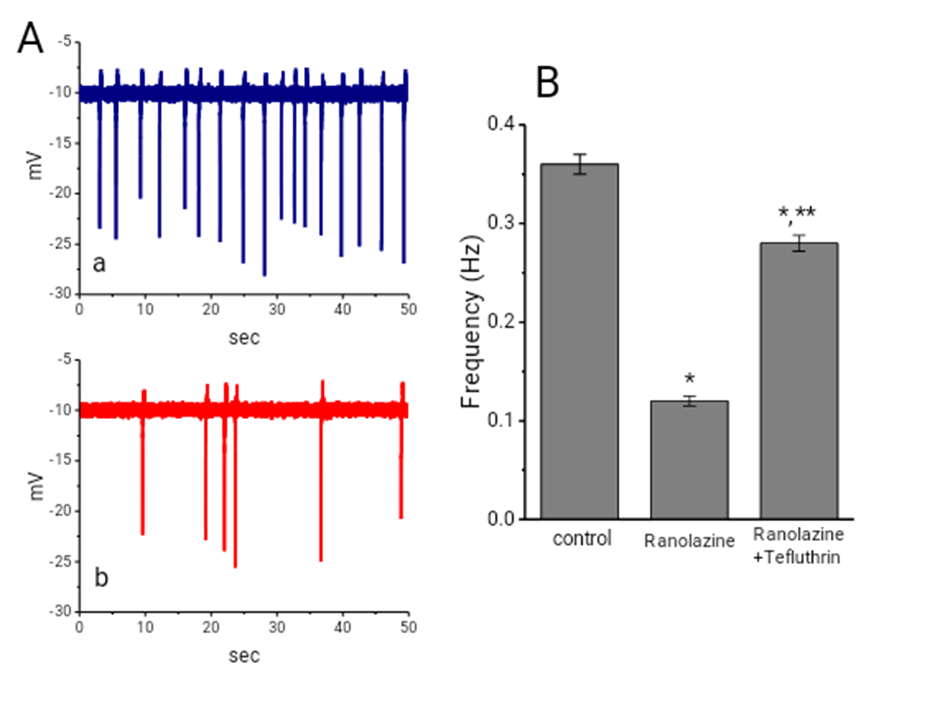

Supplement: Supplementary file 1 — Supplemental figures [file 41420_2019_143_MOESM1_ESM.docx]
